# Transcription Factors That Convert Adult Cell Identity Are Differentially Polycomb Repressed

**DOI:** 10.1371/journal.pone.0063407

**Published:** 2013-05-01

**Authors:** Fred P. Davis, Sean R. Eddy

**Affiliations:** Janelia Farm Research Campus, Howard Hughes Medical Institute, Ashburn, Virginia United States of America; Wellcome Trust Centre for Stem Cell Research, United Kingdom

## Abstract

Transcription factors that can convert adult cells of one type to another are usually discovered empirically by testing factors with a known developmental role in the target cell. Here we show that standard genomic methods (RNA-seq and ChIP-seq) can help identify these factors, as most are more strongly Polycomb repressed in the source cell and more highly expressed in the target cell. This criterion is an effective genome-wide screen that significantly enriches for factors that can transdifferentiate several mammalian cell types including neural stem cells, neurons, pancreatic islets, and hepatocytes. These results suggest that barriers between adult cell types, as depicted in Waddington's “epigenetic landscape”, consist in part of differentially Polycomb-repressed transcription factors. This genomic model of cell identity helps rationalize a growing number of transdifferentiation protocols and may help facilitate the engineering of cell identity for regenerative medicine.

## Introduction

Cell identity has long been associated with the expression of genes required for a cell's unique functions and inactivity of genes required for other cell types [1]. Transcription factors (TFs) play a central role in regulating these expression patterns, in part through interplay with chromatin-modifying complexes that alter chromatin accessibility and activity. Transcriptional control of cell identity is important during both development (when identity is established in part through precise temporal expression of TFs) and in adult cells (where key TFs maintain cell identity). Often times, the same TFs that establish cell identity are also used for its maintenance [2].

Several chromatin-based mechanisms of gene repression play a role in regulating the expression of genes important for cell identity, including Polycomb group (PcG) silencing, DNA methylation, and heterochromatin formation [3]. In particular, PcG-silencing, associated with trimethylation of lysine-27 on histone H3 (H3K27me3), is critical for establishing and maintaining cell identity, at least in part by repressing lineage-specific TFs [4]. For example, embryonic stem cells express TFs that maintain pluripotency and PcG-silence those that contribute to differentiation [5]. We recently observed that several transcription factors (TFs) regulating the identity of Kenyon cell neurons in the adult *Drosophila* brain are expressed in these cells and are PcG-repressed in another neuronal population, the octopaminergic neurons [6]. Based on our and others' findings, we hypothesized that TFs important for cell identity can be identified in pairwise comparisons of two cell types as being more highly expressed in one cell type and more strongly H3K27me3 modified in another cell type. Repressing these key TFs in other cell types is critical, because ectopic expression of TFs that regulate cell identity has the potential to convert, or “transdifferentiate”, adult cells of one type to another [7].

Transdifferentiation has been intensely studied in recent years as a potential source of cells for regenerative medicine, with the goal of obtaining replacement cells for a diseased tissue by converting other cells from the same patient. Recent reports describe small sets of TFs that can, typically at low efficiency, transdifferentiate one adult cell type (the “source” cell type) to another (“target” cell type) by “reprogramming” the nucleus to express gene batteries characteristic of the target cell type [7]. These factors have been discovered empirically by testing pools of factors (known to play a role in the maintenance or development of the target cell type) for the smallest combination (typically 3–4 factors) that induces transdifferentiation. Here we explore whether comparison of gene expression and PcG repression profiles between a pair of source and target cell types can help identify TFs that can convert one to the other. We show by reanalysis of several published datasets that most transdifferentiation factors exhibit the same genomic signature we previously observed for regulators of *Drosophila* neuronal identity – higher expression in one cell type and stronger PcG repression in another – whereas this is not true for transcription factors in general.

## Results

We identified reports that describe TFs converting adult cells (mouse or human) of one type into another, and for which expression (RNA-seq) and histone modification (H3K27me3 ChIP-seq) data obtained from both cell types could be found in the Gene Expression Omnibus (GEO) database [8] (Table 1). In total we gathered 65 datasets (38 human, 27 mouse) from 15 individual studies and two consortium projects (ENCODE, Roadmap Epigenomics Project) (Table S1, Text S1). For three cell types (hepatocytes, cardiomyocytes, and pancreatic beta-cells) without available cell type-specific genomic data, we instead used data obtained from tissues predominantly composed of these cell types: ∼60% of liver cells are hepatocytes [9], 55–75% of heart cells are cardiomyocytes [10], and 54–75% of pancreatic islet cells are beta-cells [11].

**Table 1:** Published transdifferentiation protocols used to evaluate our genomic model of cell identity.

Source cell	Target cell	Genomic Data		Transdifferentiation Factors
		Mouse	Human	
Fibroblast	Myoblast	E P		*MyoD1* [12]
Liver	Pancreas	E P H	E P H	*Pdx1* [21]
Pancreatic islet	Liver	E P H	E P H	*Cebpa* or *Cebpb* [22]
Fibroblast	Hepatocyte	E P H	E P H	*Hnf1a* and one of: *Foxa1*, *Foxa2*, *Foxa3* [49]; *Gata4*, *Hnf1a*, *Foxa3*, knockdown p19(Arf)* [50]
Fibroblast	Cardiomyocyte	E P	E P H	*Gata4*, *Mef2c*, *Tbx5* [51]; *Gata4*, *Tbx5*, Baf60c* [52]
Fibroblast	Neuron	E P		*Pou3f2* (also known as *Brn2*), *Ascl1*, *Myt1l* [53]
Liver	Neuron	E P		*Pou3f2*, *Ascl1*, *Myt1l* [54]
Fibroblast	Neural stem cell	E P H	E P H ^+^	*Sox2*, *Pou3f2*, *Foxg1* [14]; *SOX2* [26]

We obtained genomic data (E = gene expression RNA-seq, P = Polycomb-associated H3K27me3 ChIP-seq, H = Heterochromatin-associated H3K9me3 ChIP-seq) for pairs of mouse and human tissues with published transdifferentiation protocols. Genomic datasets are listed in Table S1. Asterisks (*) mark genes that were not included in our testing, because they were not transcription factors (*Baf60c*) or were knocked-down in the protocol (*p19(Arf)*). Cross (+): two sources of data were used for human neural stem cells: cortex-derived neurospheres and *in vitro* derived neural progenitor cells. We assume that the published transdifferentiation protocols apply to both human and mouse cells.

**Figure 1:**
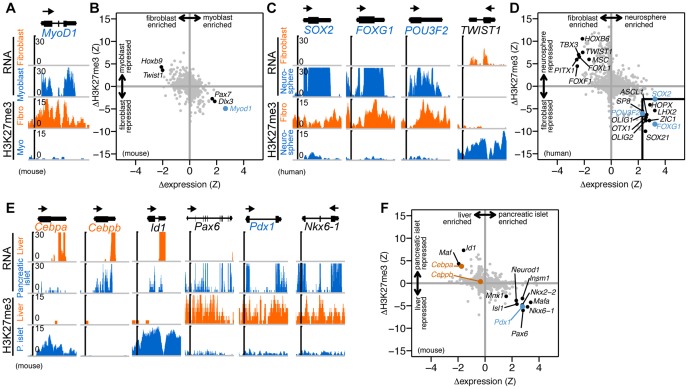
Transdifferentiation factors are more highly expressed in target cell types and more PcG repressed in source cell types. (A) Gene expression and H3K27me3 histone modification levels as measured by RNA-seq and ChIP-seq, respectively, are shown for *MyoD1*, a factor that converts fibroblasts to myoblasts [12]. Reads are displayed (units of reads per ten million mapped reads) across the *MyoD1* locus and 1 kb regions flanking the gene. The arrow above the gene structure denotes direction of transcription. Data from [55], [56]. (B) Differential expression and modification levels are shown for all transcription factors [15] (n = 1,356) annotated in the mouse genome (grey points), including *MyoD1* (blue point). (C,D) Similar plots to (A,B) are shown for factors that convert fibroblast to neural stem cells (*SOX2*, *FOXG1*, *POU3F2* [14]) and a TF with an opposite genomic pattern (*TWIST1*). The box in the lower right-hand quadrant highlights nine other TFs (black points) (of 1,447 total annotated human TFs [15]) with differential expression and modification levels similar to the transdifferentiation factors (blue points). Data from ([57], [58]) and the Roadmap Epigenomics Project (http://roadmapepigenomics.org). (E,F) Similar plots to (A, B) are shown for factors that convert liver to pancreas (*Pdx1* [21]; blue point), pancreas to liver (*Cebpa* and *Cebpb* [22]; orange points), and three other TFs with similar genomic patterns (*Id1*, *Pax6*, *Nkx6-1*; black points). Data from [59]–[61] and two other public datasets (Table S1).

We began by examining the first described transdifferentiation factor, *MyoD1*, which can convert fibroblasts to myoblasts [12]. *MyoD1* mRNA is significantly enriched in myoblasts and the gene is H3K27me3 repressed in fibroblasts, in agreement with previous reports [13] (Fig. 1A). In fact, no other TF is both more differentially expressed and more differentially H3K27me3 modified than *MyoD1* in this comparison (Fig. 1B).

Next, we examined three TFs recently shown to convert fibroblasts to neural stem cells: *SOX2*, *FOXG1*, and *POU3F2* (also known as *BRN2*) [14]. All three factors were enriched in neurospheres (a cell culture model of neural stem cells) and more H3K27me3 modified in fibroblasts (Fig. 1C). Extending this analysis to all TFs (n = 1,447) annotated [15] in the human genome, we found only 9 other factors (*ASCL1*, *HOPX*, *LHX2*, *OLIG1*, *OLIG2*, *OTX1*, *SOX21*, *SP8*, *ZIC1*) with levels of differential expression and H3K27me3 modification comparable to the known transdifferentiation factors, demonstrating 120-fold enrichment for transdifferentiation factors relative to all TFs in the genome (Fig. 1D). In contrast, using expression levels alone identified 18 other factors (69-fold enrichment) and H3K27me3 levels alone identified 36 other factors (37-fold enrichment). We obtained similar results using data measured from human neural progenitor cells derived *in vitro* from an embryonic stem cell line, with only 12 other TFs (*DMRTA1*, *FEZF1*, *LHX2*, *LIN28A*, *OTX2*, *POU3F1*, *RAX*, *SIX3*, *SOX21*, *SP8*, *ZIC2*, *ZIC5*) exhibiting a similar genomic signature to the three known transdifferentiation factors (96-fold enrichment), compared to 23 other TFs using expression alone (61-fold enrichment) and 54 other TFs using H3K27me3 alone (25-fold). In the opposite quadrant of this plot – genes that are more highly expressed in fibroblasts and more strongly H3K27me3 modified in neurospheres – we found genes that play a role in fibroblast biology (Fig. 1D). For example, *TWIST1* [16], *FOXL1* [17], and *FOXF1* [18], are implicated in “Epithelial-Mesenchymal Transition”, a transdifferentiation process where epithelial cells convert to fibroblasts. This quadrant also includes genes involved in body patterning such as *PITX1* [19] and several *HOX* genes (*HOXB6* indicated on the plot; neighboring unmarked points include *HOXB2*, *HOXB4*, *HOXB5*, *HOXB9*, and *HOXA5*) [20].

We then compared published data from pancreatic islets and liver, as transdifferentiation protocols have been described in both directions [21], [22]. We found that *Cebpa*, a factor that converts pancreatic tissue to liver cells [22], is expressed in liver cells and H3K27me3 repressed in pancreatic islets (Fig. 1E). Similarly, *Pdx1*, which converts liver tissue to pancreas [21], is highly expressed in pancreatic islets and strongly repressed in liver tissue (Fig. 1E). *Pdx1* and *Cebpa* are among the most differentially expressed and repressed transcription factors in the mouse genome in these cell types (Fig. 1F). In particular, the differential H3K27me3 modification feature significantly separates these transdifferentiation factors from other TFs with similar differential expression levels. The other factors with a similar genomic signature to *Pdx1* are all known to play a role in pancreas development, including *Nkx6-1* which promotes *Pdx1*-induced liver to pancreas transdifferentiation [23]. An apparent outlier to our expected pattern is *Cebpb*, which by itself can convert a pancreatic progenitor cell line to hepatocyte-like cells [22], but is only slightly more expressed in liver cells and slightly more repressed in pancreatic islet cells. Although *Cebpb* is typically considered a hepatic TF, it also expresses in pancreatic beta-cells, particularly under metabolic stress [24]. The transdifferentiation report that we used in our analysis was observed in a pancreatic progenitor cell line (AR42J-B13), and may not apply to pancreatic tissue *in vivo* [22]. In fact, *in vivo* overexpression of *Cebpb* in pancreatic islet cells results in pre-diabetic symptoms (reduced beta cell mass, lower plasma insulin levels, and higher glucose levels) rather than production of hepatocyte-like cells in the pancreas, suggesting that *Cebpb* may not induce transdifferentiation *in vivo* [25]. Our results predict that *Cebpa* is more likely than *Cebpb* to induce pancreas to liver transdifferentiation *in vivo*.

**Figure 2:**
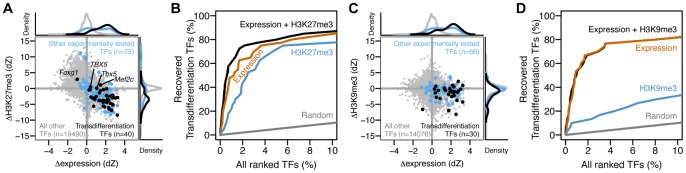
Genome-wide screening with differential expression and H3K27me3 levels significantly enriches for transdifferentiation factors. (A) We compared six human and eight mouse pairs of cell types with known transdifferentiation factors (Table 1) and plotted the differential expression (x-axis) and differential H3K27me3 modification (y-axis) for these factors (black points) as well as all other TFs in the genome (grey points). We also indicated (blue points) the subset of these other TFs that were experimentally tested for their ability to convert cell types, but not included in the conversion protocols (Table S2). Each point represents 1 gene in 1 pair of cell types. Most transdifferentiation factors are more highly expressed in the target cell (right side of the y-axis) and more highly H3K27me3 modified in the source cell (below the x-axis). The marginal distributions depict the differential expression (top) and modification (right) of transdifferentiation factors (black curve) compared to all other transcription factors (grey curve), as well as the subset that were experimentally tested (blue curve). (B) We ranked all transcription factors by differential expression (orange), differential H3K27me3 modification (blue), or a combination of both (black) and then calculated the percentage of tested transdifferentiation factors (y-axis) as a function of the position in the ranked list. (C) Plot similar to (A) showing differential H3K9me3 levels in place of H3K27me3. In contrast to H3K27me3 levels, transdifferentiation factors exhibit on average only a slight differential H3K9me3 modification. Six human and four mouse pairs of cell types were used in this analysis, as H3K9me3 modification profiles were not available for three mouse tissues (myoblast, heart, neuron; Table 1). (D) H3K9me3 modification provides minimal information beyond expression for identifying transdifferentiation factors. Data sources are listed in Table S1.

Including the examples above, in total we conducted this analysis on six human and eight mouse pairs of cell types with published transdifferentiation protocols and available genomic data (Table 1). We found that most TFs used in transdifferentiation protocols (36 of 40) are both more highly expressed in the target cell (mean dZ-expression = 1.8) and more PcG-repressed in the source cell (mean dZ-H3K27m3 = −3.1) (Fig. 2A). In contrast, all other TFs exhibit neither differential expression (mean dZ-expression = 0.02) nor differential modification (mean dZ-H3K27m3 = 0.2). The differences between transdifferentiation factors and all other TFs are highly significant (one-sided Kolmogorov-Smirnov (KS) test, expression p-value<2*10^−16^; H3K27me3 p-value<2*10^−16^). We also analyzed TFs that were experimentally tested for their ability to convert cell types but were not included in the reported protocols (Table S2, Text S1). We found that these experimentally tested TFs were significantly less differentially expressed (mean dZ-expression = 1.1, p-value = 4.3*10^−3^) and Polycomb repressed (mean dZ-H3K27m3 = −1.3, p-value = 8.0*10^−4^) than reported transdifferentiation factors (Fig. 2A). These differences are subtler than those between transdifferentiation factors and all other TFs, but are nonetheless significant and support our hypothesis. This result indicates that empirically screening a set of TFs for their ability to convert cell types identifies a subset that are, on average, more differentially expressed and repressed than the rest.

Four transdifferentiation factors (of 40 tested) did not follow our hypothesis (Fig. 2A). These exceptions fall into two groups. First, mouse *Foxg1* is both more highly expressed and less H3K27me3 modified in the source cell (fibroblast) than target cell (neural progenitor cell); In contrast, the human *FOXG1* conforms to our hypothesis (Fig. 1C,D). *Foxg1* expression in mouse embryonic fibroblasts is not unique to the RNA-seq data we used, as this has also been observed in independent microarray studies (*eg*, NCBI GEO GSE37859). This expression suggests that *Foxg1* may not be required for embryonic fibroblast conversion to neural stem cells, in line with a recent report that *SOX2* alone can also induce this conversion [26]. The second group of outliers (human *TBX5*; mouse *Tbx5*, *Mef2c*) is both more highly expressed and more H3K27me3 modified in the target cell (heart tissue as a proxy for cardiomyocytes) than source cell (fibroblasts) (Fig. 2A). The seemingly inconsistent genomic signals for these genes might be caused by their expression in a subset of heart cells (*eg*, cardiomyocytes) and repression in another subset of cells (*eg*, cardiac fibroblasts, endothelial cells), resulting in detection of both mRNA and H3K27me3 modification from the mixed tissue. Another possible explanation is that the RNA-seq data used in our analysis was measured from fetal heart tissue while the ChIP-seq data was from adult tissue (Table S1). Although *Tbx5* expression persists in the adult heart [27], *Mef2c* expression begins to decrease during embryogenesis [28]. These differences in fetal and adult expression could also contribute to the observed outlier genes.

We next asked how useful differential expression and H3K27me3 modification could be as a genome-wide screen for TFs that can transdifferentiate cells. We found that the combination of both features is more informative than either expression or H3K27me3 alone for ranking transdifferentiation factors higher than other TFs (Fig. 2B). For example, ranking with both features recovered 76% of transdifferentiation factors in the first 2% of the ranked TFs, compared to 62% by expression alone and 42% by H3K27me3 alone. Although the absolute differences in the predictive abilities of these features vary with the position in the ranked TF list, the relative order of the features is consistent (Fig. 2B).

Recently, a histone modification indicative of repressive heterochromatin, H3K9me3, was shown to regulate the identity of T helper cells [29] (Fig. 2C). We asked whether this modification could provide additional power in identifying transdifferentiation factors. The difference in H3K9me3 levels over transdifferentiation factors (mean dZ-H3K9me3 = −1.6) compared to non-transdifferentiation factors (mean dZ-H3K9me3 = −0.1) was lower in both magnitude and significance (KS-test p-value = 4.1*10^−4^) than H3K27me3 levels. Further, when used as a classifier, H3K9me3 provided no additional information beyond differential expression (Fig. 2D). These results suggest that heterochromatin (as quantified by genic H3K9me3 modification) is less important than PcG repression for transdifferentiation.

**Figure 3:**
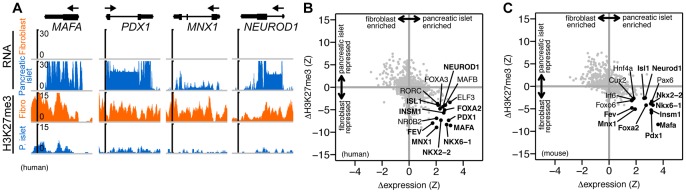
Similar genes are differentially expressed and PcG modified when comparing fibroblasts and pancreatic islets in both human and mouse. (A) Genome browser view of RNA-seq and ChIP-seq data over several genes that are more highly expressed in human pancreatic islet cells and more strongly H3K27me3 modified in fibroblasts. Axes similar to Figure 1A. (B,C) Similar sets of genes are differentially expressed and modified when comparing fibroblasts and pancreatic islets in both human and mouse. Plots similar to Figure 1B. Bold labels denote genes shown on both human and mouse plots. Data from [57]–[64], the Illumina Human Bodymap (http://www.illumina.com), the Roadmap Epigenomics Project (http://roadmapepigenomics.org), and two other public datasets (Table S1).

This analysis also enables prediction of candidate transdifferentiation factors for pairs of cell types where these are unknown. As an example, we compared fibroblasts to pancreatic islets using published data from both human and mouse tissue (Fig. 3A–C). This analysis recovered several factors shown to transdifferentiate pancreatic exocrine cells to beta-cells (*Pdx1*, *Mafa*, *Neurod1*) [30] and also identifies several others that are known to play a role in pancreatic development (*Fev*, *Pax6*, *Nkx6-1*, *Nkx2-2*, *Isl1*, *Insm1*, and *Mnx1*). The analysis identified similar factors in both human and mouse data, consistent with previous studies demonstrating conservation of cell identity regulators over long evolutionary distances [2] (Fig. 3B, 3C).

**Figure 4:**
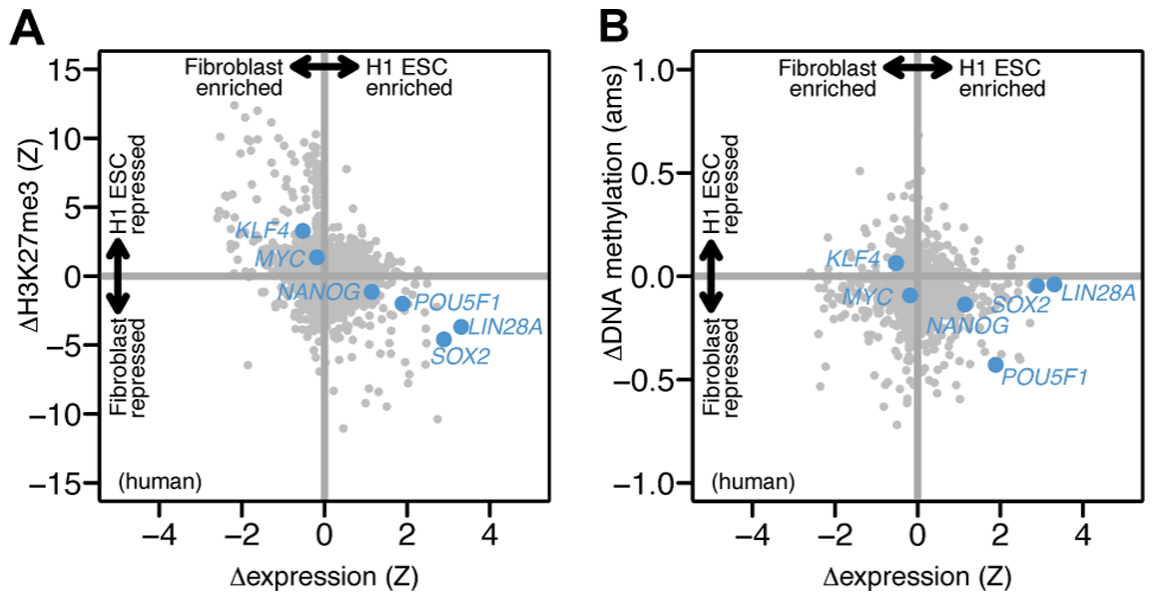
Reprogramming factors *SOX2* and *POU5F1* are PcG repressed and DNA methylated, respectively, in fibroblasts. We compared PcG repression and DNA methylation in fibroblasts and an embryonic stem cell line (H1 ESC). (A) Of the six original reprogramming factors [31], [33] (labeled points), *SOX2* is the most significantly PcG repressed in fibroblasts. Plot layout similar to Figure 1B. (B) In contrast, *POU5F1* is the most differentially methylated reprogramming factor. Differential methylation is shown in units of absolute methylation score (ams). Data from [57], [58] and the Roadmap Epigenomics Project (Table S1).

Having established the utility of differential PcG repression for identifying transdifferentiation factors, we were curious whether this pattern could also identify reprogramming factors, which induce pluripotency in adult cells [31], [32]. Two original reprogramming protocols each used four factors (*POU5F1* (also known as *OCT4*), *SOX2*, *KLF4*, *MYC* (also known as *C-MYC*) [31]; *POU5F1*, *SOX2*, *LIN28*, *NANOG* [33]). Only two factors are essential: *SOX2* and *POU5F1*. Comparing adult fibroblasts to the H1 embryonic stem cell (ESC) line, we found that *SOX2* was significantly PcG repressed in fibroblasts and expressed in the ESC (Fig. 4A). Contrary to our expectations, *KLF4* and *C-MYC* were both more repressed in ESC and more expressed in fibroblasts; however, both factors are dispensable for reprogramming [34]. *POU5F1* (a core pluripotency factor required in all reprogramming protocols) was only slightly PcG repressed in fibroblasts. This finding is a counter-example to our hypothesis and is consistent with previous reports that *POU5F1* is repressed in adult tissues by DNA methylation rather than PcG repression [5].

We next asked whether an analysis of differential DNA methylation might improve our ability to identify reprogramming factors. In contrast to H3K27me3, DNA methylation can correlate with either lower or higher gene expression depending on where it occurs in the gene structure [35]. Although this relationship is not fully established, methylation in promoters generally correlates with lower gene expression. Comparing promoter methylation in human fibroblasts and H1 ESC (as measured by published methylated DNA-immunoprecipitation (meDIP) data), we found that no other TF is both as differentially methylated and expressed as *POU5F1* (Fig. 4B). In contrast, *SOX2* was not differentially methylated. Consistent with previous work, these results suggest that DNA methylation and PcG repression both play a role in reprogramming. As more methylation data is measured and its relationship to gene expression is more precisely elucidated, differential methylation analysis may also complement Polycomb analysis for identifying transdifferentiation factors.

## Discussion

Our results demonstrate that the combined criteria of (i) greater H3K27me3 modification in the source cell and (ii) higher expression in the target cell is an effective genome-wide screen that significantly enriches for transdifferentiation factors. These observations are consistent with studies implicating PcG-silencing through H3K27me3 histone modification, for example finding roles for the H3K27me3 demethylase *Utx* [36], H3K27me3 methyltransferase *Ezh2* [37], and other PcG proteins and chromatin modifying factors in reprogramming [38]. More generally, PcG mutations affect cell identity across a broad phyletic range. For example, PcG mutations induce trans-determination in *Drosophila*, a transdifferentiation-related phenomenon where imaginal discs change their determined lineage [39].

Our results suggest that candidate transdifferentiation factors can be identified using genome-wide expression and chromatin profiles and without prior knowledge of their functional or developmental role. This approach is possible because TFs with the ability to convert the identity of a cell appear to be strongly PcG-repressed in other cell types (Fig. 2A). This repression makes intuitive sense as incorrect expression of TFs that can alter cell identity, many of which may be amplified through positive autoregulatory feedback [2], [40], could be catastrophic for the identity of other cell types.

**Figure 5:**
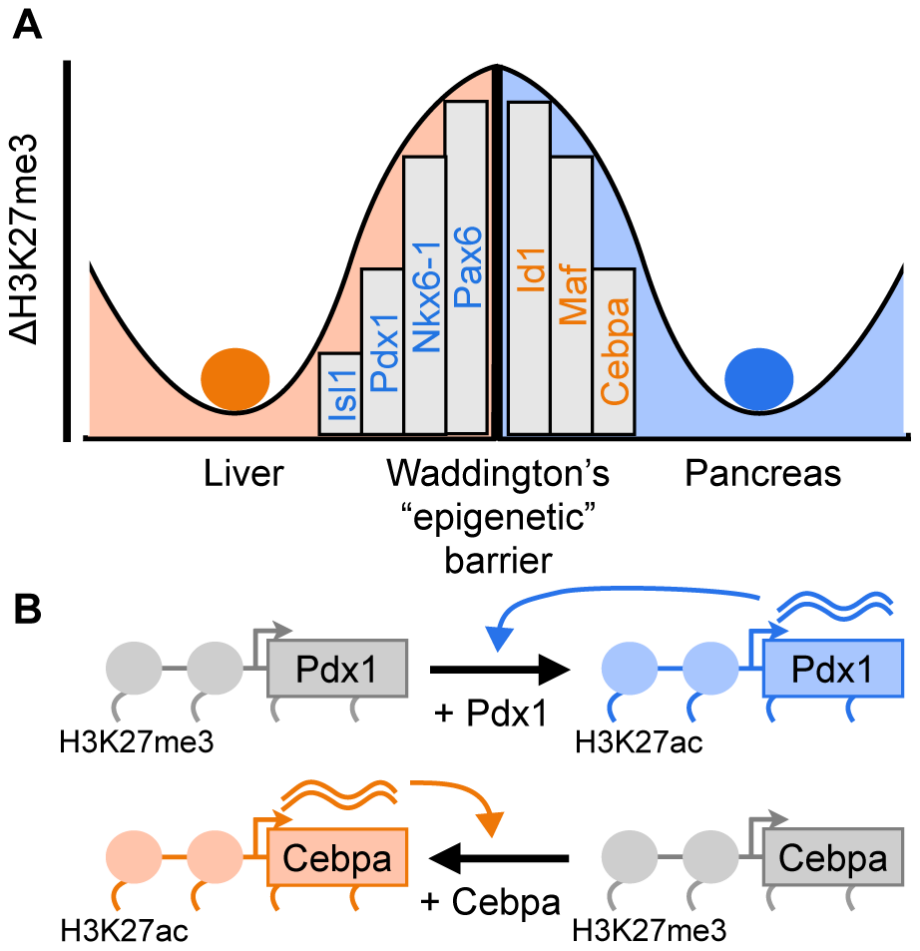
PcG repression of key transcription factors form epigenetic barriers between adult cell types. (A) Our results suggest that PcG repression of key transcription factors help form the barriers between adult cell types, as depicted by Waddington's epigenetic landscape [41]. Conceptual model based on Fig. 1F. (B) Ectopic expression of endogenously repressed transcription factors overcomes these barriers to convert one cell type to another. For example, expressing *Pdx1* in liver cells, where it is PcG repressed, converts them to pancreatic islet cells [21], where it is expressed. Expressing *Cebpa* in pancreatic islet cells, where it is PcG repressed, converts them to liver cells [22], where it is expressed. Positive autoregulation of transdifferentiation factors would stabilize the newly converted cell identity [7].

Following our results, we propose an intuitive model of cell identity where chromatin repression of key TFs acts as barriers between cell types, akin to the “epigenetic barriers” proposed by Waddington [41] (Fig. 5A). This model helps rationalize published transdifferentiation protocols and serves as an organizing framework for perturbing cell identity. First, these PcG barriers can be overcome by providing exogenous copies of TFs to a cell where it is endogenously repressed, inducing expression of its target gene batteries and where it might also induce endogenous expression of these TFs through positive autoregulatory feedback [7] (Fig. 5B). We expect that the genes predicted by this model (*eg*, Fig. 3) are suitable for testing as candidate transdifferentiation factors. These candidates may also be useful for pairs of cell types with known transdifferentiation factors, as these new genes might improve the efficiency of this typically slow and inefficient process. Second, transdifferentiation can also be induced by silencing genes, such as those that repress the gene batteries of a target cell type [42]. We did not explicitly test these factors as relatively fewer of these have been described. However, our model predicts that this class of genes is more highly expressed in the source cell and more strongly PcG-repressed in the target cell (the opposite pattern compared to “positive” transdifferentiation factors).

Although critical, identifying the appropriate transcription factors is only a part of developing an efficient transdifferentiation protocol. Empirical optimization remains important for identifying the most efficient subset of the candidate factors, their stoichiometry, timescale of induction, and the mix of growth factors and other media components to support transdifferentiation. Nevertheless, the genomic analysis we describe above should help to reduce the number of transcription factors to be tested when developing a transdifferentiation protocol.

Beyond transdifferentiation, we conjecture that TFs identified by our approach also function during normal development as “terminal selector” genes, which establish and maintain cell identity [2]. Although terminal selector genes typically refer to positive regulators of a cell's gene battery, in the context of our analysis some of these genes could also play a negative role in repressing an alternative cell type's gene battery. Following its ability to identify transdifferentiation factors, we expect that expression and chromatin profiling could also be complementary to approaches such as genetic screens that are currently used to identify terminal selector genes [2].

Our model is of course over-simplified, as expression and H3K27me3 modification levels do not capture all the mechanisms that regulate cell identity. For example, our model does not explicitly consider heterochromatin, DNA methylation, or RNA-mediated silencing. However, at least in part, expression and H3K27me3 levels may implicitly capture the effects of these other mechanisms, as these distinct systems are all interrelated in complex ways that are not yet fully characterized [3]. Nevertheless, our genomic model of cell identity helps rationalize a growing number of transdifferentiation protocols into a common framework of chromatin biology and further emphasizes the role of gene repression in cell identity. Together with higher resolution measurements enabled by new cell type-specific genomic profiling methods [6], the proposed model may facilitate engineering of cell identity for regenerative medicine.

## Methods

### RNA-seq data processing

Genomic datasets were obtained from the Gene Expression Omnibus [8] or Sequence Read Archive [43] databases (Table S1). RNA-seq data was obtained in either FASTQ (unaligned) or BED (aligned) formats. BED files were converted to FASTA format before analysis (using BEDTOOLS getfasta [44]). RNA-seq reads were processed using RSEM [45] (options “-p 8 -output-genome-bam -fragment-length-mean 250 -fragment-length-sd 50”) to estimate expression levels of all genes annotated in the iGenomes resource for the mouse mm9 and human hg19 genomes (http://cufflinks.cbcb.umed.edu/igenomes.html). To facilitate comparison between cell types, we transformed the expression levels to a logarithmic scale (log(1 + Transcripts Per Million)), and then converted these to Z-scores (number of standard deviations away from the mean). To compare expression levels between two cell types, we subtracted the corresponding Z-scores for each gene.

### ChIP-seq data processing

ChIP-seq data was analyzed as previously described [6]. Briefly, H3K27me3 and H3K9me3 ChIP-seq datasets were obtained in either aligned (BED, ELAND) or unaligned (FASTQ, SRA) formats. Aligned datasets were converted to the BAM alignment format before analysis (using BEDTOOLS bedtobam). Unaligned ChIP-seq reads were aligned to the mouse (mm9) or human (hg19) genomes using BOWTIE [46] (v 0.12.7; options “-S -t -p 8 -m 1”). We counted the number of ChIP-seq reads over each gene body (using BEDTOOLS coverageBed; options “-counts -split”), normalized this count by the gene length, and then computed a Z-score of the log(normalized counts) for each gene. This Z-score was corrected by subtracting the corresponding Z-score computed from an un-enriched input library. To compare modification levels between two cell types, we subtracted the corresponding input-corrected Z-scores for each gene. For genes with multiple isoforms, we used the isoform with the highest differential modification level.

### DNA methylation data processing

We quantified methylation over the promoters (1 kb upstream, 0.5 kb downstream of transcription start site) of all genes by analyzing methylated DNA immunoprecipitation (meDIP) data using MEDIPS [47] with recommended parameters. To quantify promoter methylation levels, we rescaled the MEDIPS absolute methylation score (AMS) to range from 0 (no methylation) to 1 (completely methylated). To compare promoter methylation levels between two cell types, we subtracted the corresponding rescaled AMS values for each gene. For genes with multiple isoforms, we used the isoform with the highest differential methylation level.

### Evaluating the genomic screens for transdifferentiation factors

Lists of transcription factors encoded in the mouse and human genomes were obtained from AnimalTFDB [15]. To evaluate the ability of differential expression or differential histone modification level to enrich for transdifferentiation factors, we ranked all TFs by these individual features and then counted the fraction of known transdifferentiation factors recovered throughout the ranked list (Fig. 2B, 2D). To evaluate the performance of the combination of expression and each histone modification, we ranked all TFs by the number of other TFs with both greater differential expression and lower differential histone modification, and counted what fraction of these other TFs were known transdifferentiation factors. To resolve ties in these ranking schemes, we applied a simple operation to all recovery curves (Fig. 2B, 2D). In cases where multiple positions in the ranked TF list recovered the same fraction of transdifferentiation factors, we used only the most highly ranking position. This procedure effectively results in smoothed recovery curves (Fig. 2B, 2D).

### Visualization and statistical analysis

ChIP-seq reads were extended to 200 nucleotides (using BEDTOOLS slopBed), the number of extended reads over all genomic positions counted (using BEDTOOLS genomeCoverageBed), and these counts visualized by IGV [48] (Fig. 1, 3). Plotting and statistical analysis was performed with the R package (http://r-project.org). Distributions of differential expression levels and histone modification levels (Fig. 2A, 2C) were compared using the one-sided Kolmogorov Smirnov test as implemented in the R ks.test() function.

## Supporting Information

Table S1 **Genomic datasets analyzed.**
(DOC)

Table S2 **Experimentally tested genes not included in transdifferentiation protocols.**
(DOC)

Text S1 **Supporting references.**
(DOC)
