# Supplementary material for: Transcription Factors That Convert Adult Cell Identity Are Differentially Polycomb Repressed
Source: PLoS One. 2013 May 1;8(5):e63407. doi: 10.1371/journal.pone.0063407 (PMC3641127; doi:10.1371/journal.pone.0063407)
Supplement: Table S1 — Genomic datasets analyzed. (DOC) [file pone.0063407.s001.doc]

**Supporting Information: Transcription factors that convert adult cell identity are differentially Polycomb repressed**

Fred P. Davis and Sean R. Eddy

Janelia Farm Research Campus,

Howard Hughes Medical Institute

19700 Helix Dr., Ashburn, VA 20147

Table S1. Genomic datasets analyzed

| **Species** | **Tissue** | **RNA-seq** | **H3K27me3 ChIP-seq** | **H3K9me3 ChIP-seq** | **ChIP-seq input** |
| --- | --- | --- | --- | --- | --- |
| Human | Fibroblast | GSM759892 [1] (foreskin) | GSM733745 [2] (dermal fibroblast) | GSM1003531:SRA [2]  GSM1003553:SRA [2]  (lung fibroblast) | GSM733750 [2]  (dermal fibroblast) |
|  |  | OTHER: Methylated DNA-immunoprecipitation (meDIP): GSM707021*:BED (foreskin) | | | |
| Human | H1 Embryonic Stem Cell (ESC) line | GSM484408*:BED | GSM434776* | GSM605328*:BED  GSM605325*:BED  GSM605327*:BED  GSM450266*:BED | GSM605338* |
|  |  | OTHER: meDIP: GSM543016*:BED | | | |
| Human | Heart (fetal) | GSM759501+ (adult) | GSM621450*:BED | GSM621434*:BED | GSM621396*:BED |
| Human | Liver | GSM759504+ | GSM537698*:BED GSM537707*:BED | GSM537710*:BED GSM537695*:BED | GSM669910*:BED GSM670008*:BED |
| Human | Neurosphere (cortex) | GSM751272*:BED | GSM707006*:BED | GSM707005*:BED | GSM707014*:BED |
| Human | Neural progenitor cell | GSM706048* | GSM818033* | GSM818056*:BED GSM818055*:BED | GSM818063*:BED |
| Human | Pancreatic islet | GSM865291:BED [3] | GSM537658*:BED | GSM537686*:BED | GSM537659*:BED |
| Mouse | Fibroblast | GSM687308 [4] | GSM640756 [5] | GSM531813 (NP1) | GSM640770 [5] |
| Mouse | Heart | GSM728909 [6] (left ventricle) | GSM742286:ELAND [7] (fetal E12.5 heart apex) | - | GSM742288:ELAND [7] (fetal E12.5 heart apex) |
| Mouse | Liver | GSM763474 [8] | GSM751034 (NP2) | GSM751035 (NP2) | GSM594590 [9] |
| Mouse | Myoblast (C2C12) | GSM628028 [10] | GSM721294 [11] | - | GSM721308 [11] |
| Mouse | Neural progenitor cell | GSM632014 [12] | GSM632042 [12] GSM632043 [12] GSM632044 [12] | GSM307616 [13] | - used ESC: GSM686996 [4] |
| Mouse | Neuron | GSM632015 [12] | GSM632051 [12] GSM632052 [12] GSM632053 [12] | - | - used ESC, listed above |
| Mouse | Pancreatic islet | GSM543647 [14] | GSM751038 (NP2) | GSM751033 (NP2) | - used liver, listed above |

Gene Expression Omnibus (GEO) [15] sample identifiers are listed for all genomic datasets analyzed in this paper. ChIP-seq input refers to un-enriched control samples. All datasets are publicly available and not under embargo. Data was downloaded from GEO (http://www.ncbi.nlm.nih.gov/geo) or the Sequence Read Archive [16] (http://trace.ddbj.nig.ac.jp) as FASTQ sequence files, unless another format (BED, ELAND, SRA) is specified. Asterisks (*) mark Roadmap Epigenomics Project data (http://www.roadmapepigenomics.org/). Plus signs (+) denote Illumina Human Body Map Project data (http://www.illumina.com). Public data was used from two studies that are not associated with a paper: GEO series GSE21271 (denoted NP1) and GSE30298 (denoted NP2). References listed in Supporting Text S1.
