# Supplementary material for: Transcription Factors That Convert Adult Cell Identity Are Differentially Polycomb Repressed
Source: PLoS One. 2013 May 1;8(5):e63407. doi: 10.1371/journal.pone.0063407 (PMC3641127; doi:10.1371/journal.pone.0063407)
Supplement: Table S2 — Experimentally tested genes not included in transdifferentiation protocols. (DOC) [file pone.0063407.s002.doc]

**Supporting Information: Transcription factors that convert adult cell identity are differentially Polycomb repressed**

Fred P. Davis and Sean R. Eddy

Janelia Farm Research Campus,

Howard Hughes Medical Institute

19700 Helix Dr., Ashburn, VA 20147

Table S2. Experimentally tested genes not included in transdifferentiation protocols.

| **Source cell** | **Target cell** | **Protocol** | **Genes tested but not included in transdiffererentiation protocols** |
| --- | --- | --- | --- |
| Fibroblast | Myoblast | [17] | MyoA*, MyoH* |
| Liver | Pancreas | [18] | - |
| Pancreatic islet | Liver | [19] | - |
| Fibroblast | Hepatocyte | [20] | *Hhex*, *Gata4+*, *Gata6*, *Tbx3*, *Hnf1a+*, *Hnf1b*, *Onecut1*, *Cebpa* |
| Fibroblast | Hepatocyte | [21] | *Foxa1+*, *Foxa2+*, *Hnf4a+*, *Onecut1*, *Hlf*, *Hhex*, *Jarid2*, *Nr2f1*, *Nr5a2*, *Nr1h4*, *Nr1i2* |
| Fibroblast | Cardiomyocyte | [22] | *Hopx, Nkx2-5, Hey2, Pitx2, Smyd1*, Myocd*, Baf60c*, Srf, Isl1, Hand2, Mesp1* |
| Fibroblast | Cardiomyocyte | [23] | Gata1, Baf60a*, Baf60b* |
| Fibroblast | Neuron | [24] | *Pou3f4, Myc, Dlx1, Hes5, Id1, Id4, Klf4, Lhx2, Mef2c, NeuroD1, Nhlh1, Nr2f1, Olig2, Pax6, Sox2, Zic1* |
| Liver | Neuron | [25] | - |
| Fibroblast | Neural stem cell | [26] | *Lhx2, Id4, Rfx4, Zic1, Dlx1, Ascl1, Olig2, Pax6* |
| Fibroblast | Neural stem cell | [27] | *BMI1*, NR2E1, HES1, POU2F1* |

We gathered lists of transcription factors (TFs) that were experimentally tested for their ability to convert cell types (either alone or in pools) and were not included in the reported transdifferentiation protocols. Dashes (-) indicate manuscripts that only described successfully tested factors. Asterisks (*) mark genes that were not included in our testing because they are not annotated (*MyoA*), are not transcription factors (*MyoH*, *Baf60c, Baf60a, Baf60b, Myocd, BMI1*) or are not annotated as transcription factors in the AnimalTFDB database [28] (*Smyd1*). Crosses (+) mark TFs that were not included in our testing because they were used in alternative protocols (also included in the table). We assume that these screen results apply to both human and mouse cells. References listed in Supporting Text S1.
