# Supplementary material for: Transcription Factors That Convert Adult Cell Identity Are Differentially Polycomb Repressed
Source: PLoS One. 2013 May 1;8(5):e63407. doi: 10.1371/journal.pone.0063407 (PMC3641127; doi:10.1371/journal.pone.0063407)
Supplement: Text S1 — Supporting references. (DOC) [file pone.0063407.s003.doc]

**Supporting Information: Transcription factors that convert adult cell identity are differentially Polycomb repressed**

Fred P. Davis and Sean R. Eddy

Janelia Farm Research Campus,

Howard Hughes Medical Institute

19700 Helix Dr., Ashburn, VA 20147

**SUPPLEMENTARY REFERENCES**

1. Cabili MN, Trapnell C, Goff L, Koziol M, Tazon-Vega B, et al. (2011) Integrative annotation of human large intergenic noncoding RNAs reveals global properties and specific subclasses. Genes Dev 25: 1915–1927. doi:10.1101/gad.17446611.

2. Consortium TEP (2012) An integrated encyclopedia of DNA elements in the human genome. Nature 489: 57–74. doi:10.1038/nature11247.

3. Eizirik DL, Sammeth M, Bouckenooghe T, Bottu G, Sisino G, et al. (2012) The human pancreatic islet transcriptome: expression of candidate genes for type 1 diabetes and the impact of pro-inflammatory cytokines. PLoS Genet 8: e1002552. doi:10.1371/journal.pgen.1002552.

4. Lienert F, Mohn F, Tiwari VK, Baubec T, Roloff TC, et al. (2011) Genomic prevalence of heterochromatic H3K9me2 and transcription do not discriminate pluripotent from terminally differentiated cells. PLoS Genet 7: e1002090. doi:10.1371/journal.pgen.1002090.

5. Koche RP, Smith ZD, Adli M, Gu H, Ku M, et al. (2011) Reprogramming factor expression initiates widespread targeted chromatin remodeling. Cell Stem Cell 8: 96–105. doi:10.1016/j.stem.2010.12.001.

6. Lee J-H, Gao C, Peng G, Greer C, Ren S, et al. (2011) Analysis of transcriptome complexity through RNA sequencing in normal and failing murine hearts. Circ Res 109: 1332–1341. doi:10.1161/CIRCRESAHA.111.249433.

7. He A, Ma Q, Cao J, Von Gise A, Zhou P, et al. (2012) Polycomb repressive complex 2 regulates normal development of the mouse heart. Circ Res 110: 406–415. doi:10.1161/CIRCRESAHA.111.252205.

8. Yu C, Li Y, Holmes A, Szafranski K, Faulkes CG, et al. (2011) RNA sequencing reveals differential expression of mitochondrial and oxidation reduction genes in the long-lived naked mole-rat when compared to mice. PLoS ONE 6: e26729. doi:10.1371/journal.pone.0026729.

9. Creyghton M, Cheng A, Welstead G, Kooistra T, Carey B, et al. (2010) Histone H3K27ac separates active from poised enhancers and predicts developmental state. Proc Natl Acad Sci USA. Available:http://www.pnas.org/content/107/50/21931.long.

10. Mousavi K, Zare H, Wang AH, Sartorelli V (2012) Polycomb protein Ezh1 promotes RNA polymerase II elongation. Mol Cell 45: 255–262. doi:10.1016/j.molcel.2011.11.019.

11. Asp P, Blum R, Vethantham V, Parisi F, Micsinai M, et al. (2011) Genome-wide remodeling of the epigenetic landscape during myogenic differentiation. Proc Natl Acad Sci USA 108: E149–158. doi:10.1073/pnas.1102223108.

12. Tiwari VK, Stadler MB, Wirbelauer C, Paro R, Schübeler D, et al. (2012) A chromatin-modifying function of JNK during stem cell differentiation. Nat Genet 44: 94–100. doi:10.1038/ng.1036.

13. Mikkelsen TS, Ku M, Jaffe DB, Issac B, Lieberman E, et al. (2007) Genome-wide maps of chromatin state in pluripotent and lineage-committed cells. Nature 448: 553–560. doi:10.1038/nature06008.

14. Kim H, Toyofuku Y, Lynn FC, Chak E, Uchida T, et al. (2010) Serotonin regulates pancreatic beta cell mass during pregnancy. Nat Med 16: 804–808. doi:10.1038/nm.2173.

15. Barrett T, Troup DB, Wilhite SE, Ledoux P, Evangelista C, et al. (2011) NCBI GEO: archive for functional genomics data sets--10 years on. Nucleic Acids Res 39: D1005–1010. doi:10.1093/nar/gkq1184.

16. Kodama Y, Shumway M, Leinonen R (2012) The Sequence Read Archive: explosive growth of sequencing data. Nucleic Acids Res 40: D54–56. doi:10.1093/nar/gkr854.

17. Davis R, Weintraub H, Lassar A (1987) Expression of a single transfected cDNA converts fibroblasts to myoblasts. Cell 51: 987–1000.

18. Ber I, Shternhall K, Perl S, Ohanuna Z, Goldberg I, et al. (2003) Functional, persistent, and extended liver to pancreas transdifferentiation. J Biol Chem 278: 31950–31957. doi:10.1074/jbc.M303127200.

19. Burke ZD, Shen C-N, Ralphs KL, Tosh D (2006) Characterization of liver function in transdifferentiated hepatocytes. J Cell Physiol 206: 147–159. doi:10.1002/jcp.20438.

20. Sekiya S, Suzuki A (2011) Direct conversion of mouse fibroblasts to hepatocyte-like cells by defined factors. Nature 475: 390–393. doi:10.1038/nature10263.

21. Huang P, He Z, Ji S, Sun H, Xiang D, et al. (2011) Induction of functional hepatocyte-like cells from mouse fibroblasts by defined factors. Nature 475: 386–389. doi:10.1038/nature10116.

22. Ieda M, Fu J-D, Delgado-Olguin P, Vedantham V, Hayashi Y, et al. (2010) Direct reprogramming of fibroblasts into functional cardiomyocytes by defined factors. Cell 142: 375–386. doi:10.1016/j.cell.2010.07.002.

23. Takeuchi JK, Bruneau BG (2009) Directed transdifferentiation of mouse mesoderm to heart tissue by defined factors. Nature 459: 708–711. doi:10.1038/nature08039.

24. Vierbuchen T, Ostermeier A, Pang ZP, Kokubu Y, Südhof TC, et al. (2010) Direct conversion of fibroblasts to functional neurons by defined factors. Nature 463: 1035–1041. doi:10.1038/nature08797.

25. Marro S, Pang Z, Yang N, Tsai M, Qu K, et al. (2011) Direct Lineage Conversion of Terminally Differentiated Hepatocytes to Functional Neurons. Cell Stem Cell 9: 374–382. doi:10.1016/j.stem.2011.09.002.

26. Lujan E, Chanda S, Ahlenius H, Südhof TC, Wernig M (2012) Direct conversion of mouse fibroblasts to self-renewing, tripotent neural precursor cells. Proc Natl Acad Sci USA 109: 2527–2532. doi:10.1073/pnas.1121003109.

27. Ring KL, Tong LM, Balestra ME, Javier R, Andrews-Zwilling Y, et al. (2012) Direct reprogramming of mouse and human fibroblasts into multipotent neural stem cells with a single factor. Cell Stem Cell 11: 100–109. doi:10.1016/j.stem.2012.05.018.

28. Zhang H-M, Chen H, Liu W, Liu H, Gong J, et al. (2012) AnimalTFDB: a comprehensive animal transcription factor database. Nucleic Acids Res 40: D144–149. doi:10.1093/nar/gkr965.
